# Building a rural medical workforce: the foundations of a place-based approach to program evaluation

**DOI:** 10.3389/fmed.2025.1582793

**Published:** 2025-06-26

**Authors:** Lara Fuller, Jessica Beattie, Vincent L. Versace, Gary D. Rogers, Matthew Richard McGrail

**Affiliations:** ^1^School of Medicine, Deakin University, Geelong, VIC, Australia; ^2^Rural Community Clinical School, School of Medicine, Deakin University, Colac, VIC, Australia; ^3^Deakin Rural Health, School of Medicine, Deakin University, Warrnambool, VIC, Australia; ^4^Rural Clinical School, The University of Queensland, Rockhampton, QLD, Australia

**Keywords:** rural medical education, rural footprint, place-based, program evaluation, longitudinal integrated clerkship, rural clinical school, rural workforce

## Abstract

**Introduction:**

To address the maldistribution of medical practitioners within Deakin University’s rural training footprint, a place-based Rural Training Stream (RTS) was established (2022). Formal definition of the footprint has enabled priority admission of 30 local students annually. This paper describes graduate workforce outcomes for the footprint, providing a baseline for future evaluation of the RTS.

**Methods:**

Graduates’ (2011–2022) Principal Places of Practice (2023) were extracted from the Australian Health Practitioner Regulation Agency register and linked with demographic, admission and training data. Descriptive statistics, univariate analysis and multinomial logistic regression were employed to describe associations with practice in three defined rural Tiers (Tier 1: Deakin’s rural footprint, Tier 2: other rural Victoria, Tier 3: other rural Australia), with metropolitan practice as the reference group.

**Results:**

120 (39.2%) graduates were working in Tier 1 and 93 (30.4%) in each of Tiers 2 and 3. Significant associations (*p* < 0.001) with working in the footprint were: post-graduate years 1–3 (OR 7.2), rural longitudinal integrated clerkship and rural clinical school (RCS) pathway (OR 6.8); RCS pathway only (OR 4.1), general practice specialty (OR 4.7) and rural background (OR 3.0).

**Discussion:**

The differential effect of rural training on graduates working in the rural footprint, compared with other parts of rural Victoria and Australia is noteworthy. Attrition of graduates from the footprint beyond post-graduate year three highlights the urgency of expanding rural specialty training pathways. These baseline data reinforce the place-based design of the RTS and provide a foundation for future evaluation of local workforce outcomes.

## Introduction

In rural areas, access to healthcare is impacted by both geographic and specialty maldistribution of health professionals, including medical practitioners (1). Medical schools have a critical role to play in addressing these challenges through the adoption of comprehensive approaches to increase the number of graduates who will go on to practice in rural communities (2). Described elements of comprehensive approaches include selection of rural background students, extended periods of rural clinical training, rurally oriented curricula and integration with post-graduate rural training pathways (2).

Increasingly, place-based approaches have been identified as a critical component of addressing the medical workforce maldistribution (3). Place-based approaches consider the unique needs, conditions and opportunities of defined communities/regions and collaboratively engage local stakeholders to address complex socioeconomic issues within a defined geographic area (3, 4). Rural medical education lends itself to a place-based approach when there is a clear relationship between a medical school and a defined region, a collaborative approach to the recruitment and training of students, and an evaluation of outcomes that includes accountability to the region’s workforce needs (5).

Deakin University’s School of Medicine, established in 2008, includes funding from the Australian Government’s Rural Health Multidisciplinary Training (RHMT) program to support its rural clinical schools (RCS) (6), with a key objective being to serve the workforce needs of Western Victoria. The primary workforce need for the region is an adequate supply of general practitioners (GPs) and rural generalists (RGs), the Australian equivalent of family medicine practitioners (7). GPs and RGs have a broad skillset and provide comprehensive primary care for individuals, families and communities (8). The School of Medicine’s rural activity footprint, formally defined in 2020, comprises rural areas of the Western Victoria Primary Healthcare Network (PHN), west of the Greater Geelong local government area (9). The GP workforce is maldistributed within the PHN, with 62.3% working in areas classified as metropolitan or regional centers (Greater Geelong and Ballarat) (10). In 2023, only 5.3% (430) of the states 8,141 GPs were working in the rural footprint, with 64.2% (276/430) of these GPs over 45 years of age (10).

Deakin’s Doctor of Medicine (MD) is a four-year graduate entry medical program. Until 2022, students meeting the national RHMT program definition of rural background were admitted to the course through an entry quota (between 25% and 30%), aided by the application of rurality bonuses during selection (11). All MD students spent the first two pre-clinical years at the Waurn Ponds (Geelong, classified metropolitan) campus, following which they completed two years of clinical training at one of five clinical schools; two metropolitan schools (Eastern Health and Geelong), and three RCS [Ballarat, Warrnambool and the Rural Community Clinical School (RCCS)]. The RCCS uniquely offers a 12-month longitudinal integrated clerkship (LIC) based in rural GP, following which students relocate to another metropolitan or RCS to complete year four.

Noting deficiencies of early graduate outcomes in the Deakin program and a desire to address the critical regional workforce shortages, a Rural Training Stream (RTS), designed to incorporate comprehensive, place-based training strategies, was established in Deakin’s MD course in 2022 (12). A key element of establishing the RTS was formally defining Deakin’s rural training footprint, enabling the priority admission of 30 local students annually (9). These applicants (Tier 1) are selected ahead of applicants from other parts of rural Victoria (Tier 2) and rural Australia (Tier 3). Moreover, having a defined footprint enables more specific evaluation of work location outcomes, which previously was largely limited to being rural or metropolitan (13).

This paper aims to describe Deakin’s graduate workforce outcomes for the rural footprint (“locally”), at the time of its RTS implementation, compared to the rest of rural Victoria and Australia, and reports on the demographic and training variables that are associated with graduates working locally. This provides baseline evidence of the regional workforce outcomes prior to the establishment of the RTS and key data for future comparison, as well as important feedback for the rural communities that have invested over an extended period in training Deakin’s students.

## Materials and methods

Deakin graduates’ (2011–2022) Principal Places of Practice (PPP) in 2023 were extracted from the Australian Health Practitioner Regulation Agency (AHPRA) register. PPPs were assigned to one of four geographical location groups, which align with the RTS selection Tiers. These are built upon Australia’s national seven-level rurality classification, the Modified Monash (MM) model which defines locations as metropolitan areas (MM1), regional centers (MM2), large rural towns (MM3), medium rural towns (MM4), small rural towns (MM5), remote communities (MM6) and very remote communities (MM7) (14):

(i) Tier 1: 965 localities within Deakin’s rural training footprint (MM2–6)
(ii) Tier 2: locations outside Deakin’s footprint, in other parts of rural Victoria (MM2–6)
(iii) Tier 3: locations outside of Victoria, in other parts of rural Australia (MM2–7) or
(iv) Tier 4: metropolitan locations (MM1).

Administrative data were extracted from the School of Medicine’s graduate database including gender, rural background (RB), bonded medical place (BMP), clinical school training pathway, and graduation year. These data were linked to graduates’ PPPs by a data manager, de-identified, and provided to the research team for analysis. Post-graduate years (PGY) were assigned to four categories for analysis, reflecting different career stages after medical school: PGY 1–3, 4–6, 7–9, and 10–12. BMPs were considered to be any form of bonded return of service schemes in which graduates were enrolled. Graduates’ registered specialties were assigned as no specialty, GP or non-GP specialty.

Four categories of clinical school training pathways were assigned:

(i) metropolitan training only (Metro)
(ii) 2 years of regional rural clinical school training (RCS)
(iii) LIC and 1 year of regional rural clinical school training (LIC/RCS)
(iv) LIC and 1 year of metropolitan clinical school training (LIC/metro).

All analyses were conducted using SPSS statistical software v30 (IBM, New York, NY, USA). Descriptive statistics, univariate analysis using Pearson’s chi square, and multinomial logistic regression modeling were employed to describe graduate workforce outcomes and variables associated with working in each of the three rural location Tiers, with metropolitan practice as the reference group. The regression model included significant univariates (*p* < 0.05), with an analysis conducted to determine odds ratios for graduates working in each of the three rural Tiers, with metropolitan work location as the reference group.

## Results

Registered Australian PPPs for 1,508 graduates were available, with 51 graduates from the 12 cohorts missing location data or outside Australia. 306 (20.3%) of graduates had a PPP in a rural location, with 155 (10.3%) in regional centers (MM2), 65 (4.3%) in large rural towns (MM3), 43 (2.9%) in medium rural towns (MM4), 33 (2.2%) in small rural towns (MM5), 6 (0.4%) in remote communities (MM6) and 4 (0.3%) in very remote communities (MM7).

**FIGURE 1:**
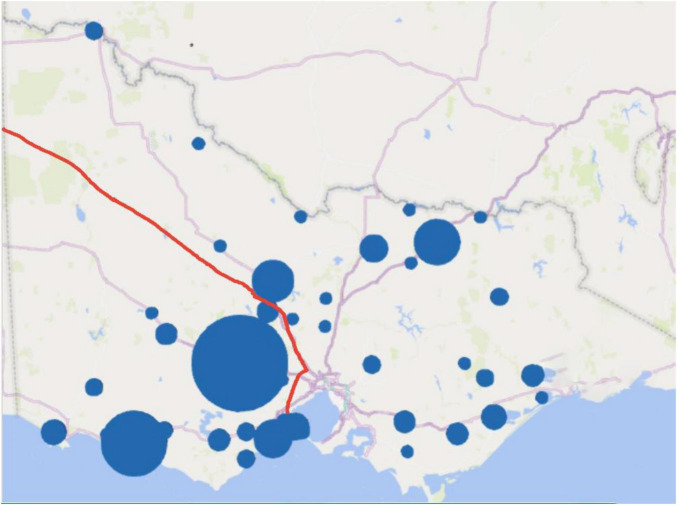
Work locations of Deakin MD graduates in rural Victoria, 2023. *Red line outlines rural footprint.

Of the 306 graduates with rural PPPs in 2023, 120 (39.2%) were working in Deakin’s rural footprint (Tier 1), and 93 (30.4%) working in each of Tiers 2 and 3 (Table 1). Within Deakin’s rural footprint, graduates were clustered around the major RCS training locations situated in a regional center (MM2—Ballarat) and a large rural town (MM3—Warrnambool), aligned with the locations of early post-graduate training positions (Figure 1).

**TABLE 1:** Univariate associations with graduates’ (2011–2022) AHPRA registered principal place of practice (2023), by Tier.

	Tier 1: rural footprint	Tier 2: other rural Victoria	Tier 3: other rural Australia	Tier 4: metropolitan	*P*-value
All graduates (*n* = 1,508)	120 (8.0%)	93 (6.2%)	93 (6.2%)	1,202 (79.7%)	
**Gender**					
Female (*n* = 745)	60 (8.1%)	44 (5.9%)	41 (5.5%)	600 (80.5%)	0.715
Male (*n* = 763)	60 (7.9%)	49 (6.4%)	52 (6.8%)	602 (78.9%)	
**Rural background**					
No (*n* = 1,120)	68 (6.1%)	52 (4.6%)	51 (4.6%)	949 (84.7%)	< 0.001
Yes (*n* = 388)	52 (13.4%)	41 (10.6%)	42 (10.8%)	253 (65.2%)	
**Bonded medical place**					
No (*n* = 1,086)	80 (7.4%)	58 (5.3%)	64 (5.9%)	884 (81.4%)	0.049
Yes (*n* = 422)	40 (9.5%)	35 (8.3%)	29 (6.9%)	318 (75.4%)	
**Training pathway**					
Metro only (*n* = 857)	35 (4.1%)	53 (6.2%)	47 (5.5%)	722 (84.2%)	< 0.001
RCS (*n* = 451)	65 (14.4%)	21 (4.7%)	32 (7.1%)	333 (73.8%)	
LIC/metro (*n* = 142)	10 (7.0%)	9 (6.3%)	8 (5.6%)	115 (81.0)	
LIC/RCS (*n* = 58)	10 (17.2%)	10 (17.2%)	6 (10.3%)	32 (55.2%)	
**Post-graduate year group**					
PGY 1–3 (*n* = 433)	63 (14.5%)	31 (7.2%)	13 (3.0%)	326 (75.3%)	< 0.001
PGY 4–6 (*n* = 365)	20 (5.5%)	21 (5.8%)	21 (5.8%)	303 (83.0%)	
PGY 7–9 (*n* = 350)	19 (5.4%)	19 (5.4%)	33 (9.4%)	279 (79.7%)	
PGY 10–12 (*n* = 360)	18 (5.0%)	22 (6.1%)	26 (7.2%)	294 (81.7%)	
**Vocation**					
No specialty (*n* = 1,115)	91 (8.2%)	62 (5.6%)	63 (5.7%)	899 (80.6%)	< 0.001
GP (*n* = 243)	28 (11.5%)	27 (11.1%)	25 (10.3%)	163 (67.1%)	
Non-GP specialist (*n* = 150)	1 (0.7%)	4 (2.7%)	5 (3.3%)	140 (93.3%)	

Table 1 displays the univariate associations between graduates’ demographic and training variables and their practice location. In the univariate analysis, RB, BMP, clinical training pathway, PGY group and vocation had a significant association with work location (*p* < 0.05). BMP did not retain statistical significance for any rural practice Tier in the regression model.

### Rural background

Table 2 summarizes regression model results of the associations between graduates’ characteristics and work location. Graduates with a RB were working in all rural Tiers in higher proportions than metropolitan background graduates (*p* < 0.001). The respective odds ratios were Tier 1: OR 3.02 (95% CI 2.00–4.57); Tier 2: OR 2.81 (95% CI 1.80–4.37); and Tier 3: OR 2.91 (95% CI 1.87–4.50).

**TABLE 2:** Multinomial logistic regression model and odds ratios for 2011–2022 graduates’ principal place of practice (2023) in (1) Tier 1: Deakin’s rural footprint, (ii) Tier 2: other rural Victoria and (iii) Tier 3: other rural Australia.

	Tier 1: Deakin’s rural footprint		Tier 2: other rural Victoria		Tier 3: other rural Australia	
	**Odds ratio (95% CI)**	***P*-value**	**Odds ratio (95% CI)**	***P*-value**	**Odds ratio (95% CI)**	***P*-value**
**Metropolitan background (reference)**						
Rural background	3.02 (2.00–4.57)	< 0.001	2.81 (1.80–4.37)	< 0.001	2.91 (1.87–4.50)	< 0.001
**Non-bonded place (reference)**						
BMP	1.13 (0.74–1.74)	0.57	1.50 (0.95–2.36)	0.08	1.12 (0.70–1.78)	0.65
**Metropolitan training pathway (reference)**						
LIC/RCS	6.80 (2.94–15.72)	< 0.001	3.71 (1.67–8.27)	0.001	2.32 (0.90–5.96)	0.82
LIC/metro	1.55 (0.72–3.29)	0.26	0.90 (0.42–1.92)	0.79	0.94 (0.42–2.08)	0.88
RCS	4.08 (2.60–6.38)	< 0.001	0.81 (0.48–1.38)	0.44	1.40 (0.87–2.26)	0.17
**PGY 10–12 (reference)**						
PGY 1–3	7.24 (3.16–16.56)	< 0.001	2.62 (1.15–5.96)	0.02	0.46 (0.21–1.01)	0.05
PGY 4–6	1.95 (0.82–4.63)	0.13	1.69 (0.74–3.86)	0.21	0.73 (0.36–1.49)	0.39
PGY 7–9	1.29 (0.623–2.67)	0.49	1.10 (0.55–2.20)	0.79	1.22 (0.82–2.64)	0.50
**No specialty (reference)**						
GP	4.65 (2.26–9.56)	< 0.001	3.76 (1.88–7.55)	< 0.001	1.47 (0.82–2.64)	0.19
Other specialty	0.23 (0.03–1.83)	0.16	0.71 (0.22–2.37)	0.58	0.37 (0.13–1.02)	0.54

### Training pathway

All LIC and/or RCS pathways saw a higher proportion of graduates working in Tier 1 compared with Tiers 2 and 3 (Table 1). LIC/RCS graduates had 6.80 times the odds of PPP in the footprint (95% CI 2.94–15.72, *p* < 0.001) and RCS graduates had 4.08 times increased odds (95% CI 2.60–6.38, *p* < 0.001), compared with metropolitan trained graduates (Table 2). In contrast, metro pathway graduates working in a rural area were least likely to be in a Tier 1 location.

Outside Tier 1, LIC/RCS students were the only group with significantly increased odds of working rurally in other parts of rural Victoria (Tier 2) compared with metropolitan trainees (OR 3.71, 95% CI 1.67–8.27, *p* = 0.001). There were no other significant associations found in the regression analysis between any form of extended rural training and graduates working in other parts of rural Victoria or Australia, outside the rural training footprint (Tiers 2 or 3).

**FIGURE 2:**
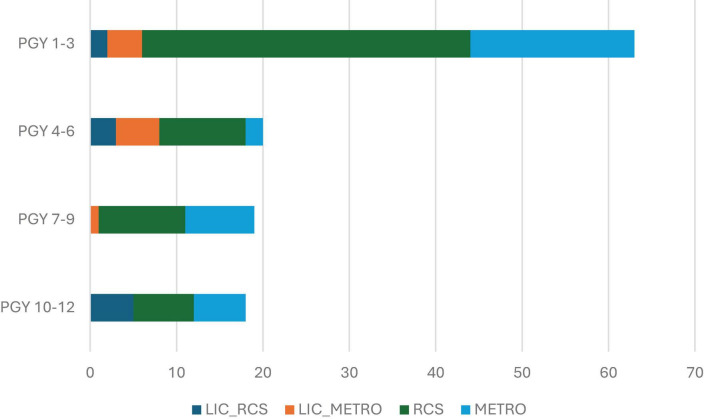
Number of graduates working in Deakin’s rural footprint (Tier 1) in 2023, by post-graduate year (PGY) and training pathway.

RCS graduates comprised the majority of graduates working in the rural footprint in every PGY group (Figure 2).

### Post-graduate year group

The overall proportion of graduates in rural practice fluctuated between PGY groups, highest at 24.7% in PGY 1–3 and lowest at 17.0% in PGY 4–6, however, the distribution of graduates between the Tiers changed significantly over time (*p* < 0.001). In the PGY 1–3 group, 14.5% of graduates had a PPP in the rural footprint compared to 5.5% in PGY 4–6. This proportion remained similarly low at 5.0% for PGY 10–12 (Table 1). There was a concomitant increase in the proportion of graduates working in metropolitan areas (from 75.3% of graduates in metropolitan areas in PGY 1–3 to 83.0% in PGY 4–6), and in other areas of rural Australia (from 3.0% in PGY 1–3 to a maximum of 9.4% in PGY 7–9). The proportion of graduates working in other areas of rural Victoria fell from 7.2% in PGY 1–3 to a low of 5.4% at PGY 5–7.

Given the cross-sectional methodology, an analysis was conducted to identify any significant differences in PGY cohort characteristics that may be impacting on these findings. There was a significant difference found in the clinical school pathways undertaken (*p* = 0.014), with a higher proportion of graduates in the PGY 10–12 cohort having undertaken the LIC/RCS pathway and fewer the LIC/metro pathway than in all subsequent PGY groups. There were no differences between the PGY groups in the presence of students with RB or BMPs.

### Vocation

There was a significant association between graduates with a specialty in GP working in the rural footprint (OR 4.65, 95% CI 2.26–9.56, *p* < 0.001) or other rural Victoria (OR 3.76, 95% CI 1.88–7.55, *p* < 0.001) compared with those with no registered specialty. This finding was not observed in other rural Australia (Table 2).

Graduates of the LIC/RCS pathway were most likely to be in GP, with 15/58 (25.9%) registered as GPs, compared with 133/857 (15.5%) metropolitan, 77/451 (17.1%) RCS and 18/142 (12.7%) LIC/metro grads.

There was particularly strong rural practice (80.0%) observed amongst the small number of LIC/RCS graduates with a GP vocation, though their distribution was similar across the rural Tiers [Tier 1: 4 (26.7%), Tier 2: 5 (33.3%), Tier 3: 3 (20.0%), Metropolitan: 3 (20.0%)].

## Discussion

As place-based health professional education programs seek to train and retain graduates in the rural communities with which they partner, an understanding of the factors that influence graduate retention in the local region is critical. The results of this study provide important insights into the foundations of an effective place-based approach to rural workforce development and set a baseline for future comparisons of Deakin’s graduate workforce outcomes.

### Place-based rural clinical training keeps graduates in the footprint

An important finding of this study is the differential effect of extended rural clinical training on graduates working in the rural footprint, particularly early in their medical career, compared with other rural areas of Victoria and Australia. There was a significant effect of 2-year extended rural training pathways on graduates working in the footprint (LIC/RCS OR 6.80; RCS OR 4.08), but a lesser association was seen with working in other parts of rural Victoria (for the LIC/RCS pathway only, OR 3.7) and no association was found with working in other parts of rural Australia. This finding is supported by other research that found a stronger effect of rural training on working in the same region, compared to other rural areas (15). Whilst this is a positive outcome for rural communities investing in student training, training capacity limitations restrict the extent to which these rural pathways can be expanded. Strategies to enhance rural workforce outcomes therefore need to align rural training with other influential elements.

The significant influence of LIC participation on graduate workforce outcomes in the rural footprint (OR 6.8) supports other literature demonstrating the important association between LIC participation and positive rural workforce outcomes, including choice of GP (16). LIC students spend substantial amounts of time learning within the GP context, which is proposed to influence their career decisions through a range of mechanisms (16, 17). However, the findings also stress the importance of rural training continuity for LIC programs, noting the significant differences in work location and vocation between those LIC students who completed a second RCS year compared with those who returned to a metropolitan clinical school. The former were more than twice as likely to work rurally (LIC/RCS 44.8%, LIC/metro 19.0%) and entered GP in greater proportions (LIC/RCS 25.9%, LIC/metro 12.7%).

### Expanded opportunities for rural post-graduate training are required

The results demonstrate stronger retention in the rural footprint during the early post-graduate years, with a significant decline evident at PGY 4–6 that was then sustained. This loss after PGY 1–3 was associated with a concomitant increase in metropolitan-based graduates, likely an indication that many graduates are leaving the rural footprint after their junior medical training (intern/resident years) to pursue specialty training in metropolitan areas during this time period (18). Similar migration patterns have been observed in longitudinal studies, postulating the lack of available training pathways, especially outside of GP, as a key contributor to rural workforce shortages due to the disruption in the continuity of rural training (19, 20). The analysis of PGY cohort characteristics suggests there is nothing specific about the more recently graduated cohorts (PGY 1–3) to explain why they would be inherently more likely to work in the footprint, in fact the opposite might be expected based on their comparatively lower completion of LIC/RCS training pathways. The greater retention in the rural footprint in the early post-graduate years is therefore likely attributable to the availability of local prevocational training opportunities (PGY 1–3) and subsequent lack of vocational training opportunities beyond PGY 3.

In Western Victoria, completion of all components of GP training within the rural footprint has been possible for all Deakin graduates. The strong retention of graduates working in GP in the footprint (OR 4.65), suggests that when training pathways are locally available, graduates are more likely to train in place and remain working in the region.

The need to provide alternative rural specialty training pathways has been identified as a key priority of Australia’s National Medical Workforce Strategy 2021–2031 and a 2024 recommendation of the Medical Deans of Australia and New Zealand (3, 18). The availability of these pathways within our footprint has been steadily growing, supported by the establishment of the Western Victoria Regional Training Hub in 2017 and the Victorian Rural Generalist Program (VRGP) in 2019. The VRGP provides support for GPs to gain advanced skills aligned with the needs of rural communities. As more rural specialty pathways including surgery, internal medicine, rural generalism, emergency medicine, pediatrics and obstetrics and gynecology are being added to the local rural training offerings, we anticipate enhanced graduate retention in the footprint beyond PGY 3 in future. Longitudinal graduate tracking studies would be beneficial to further evaluate graduate migration patterns and the influence of the development of new rural post-graduate specialty training pathways on future retention in the footprint.

### Rural background students need access to local training

It has been well-established through international research that RB students are more likely to work rurally as graduates (21–25). This is a driver for policies, such as Australia’s RHMT program, that requires all universities with a RCS to admit a minimum quota of RB students annually. However, Australia’s national rural medical school admissions system results in RB students relocating throughout the country to gain one of these competitive places in medical school. The finding that our RB graduates were similarly likely to work in each of the rural Tiers (Tier 1: OR 3.02; Tier 2 OR 2.81; Tier 3: OR 2.91) may be an indication that following completion of training, graduates return to their home regions. This movement dislocates graduates from rural communities, potentially disrupting rural training continuity at a critical time. There is a need for local admission pathways to be developed that allow rural students to have priority access to medical training in their home regions, and for national policy that supports this approach (3).

With the commencement of the RTS and the place-based recruitment of RB students from the rural footprint, our future research will report on workforce outcomes for RB graduates based on their Tier of origin, to determine whether priority admission of local rural students enhances rural workforce outcomes for the footprint beyond the general admission of RB students.

### A comprehensive place-based approach

Comprehensive approaches to rural medical training unite more than one effective element in program design to improve rural workforce outcomes (2, 26). From 2028, all Deakin RTS graduates are anticipated to have a rural background within the footprint and will also have completed four years of rural training there. Based on the evidence, this place-based alignment is anticipated to increase the proportion of Deakin’s graduates who remain working in the rural training footprint over time, if other limiting factors such as the availability of post-graduate training pathways are addressed. Future research into Deakin’s graduate workforce outcomes will investigate the effect of the RTS on outcomes for the region.

### Limitations

Given the RHMT program definition was used to identify RB graduates for the admission of these cohorts, the present study does not enable us to determine the differential workforce outcomes in relation to graduates having a RB in the rural footprint, compared with other parts of rural Australia. An analysis of the differential influence of graduates having a RB within or outside of the rural footprint will be included in our future graduate studies. The use of PPP to identify graduates working rurally does not capture all contributions that graduates may make to the rural workforce, for example through visiting services. The lower numbers of graduates completing some training pathways, or having completed their specialty training thus far, limits the confidence with which findings can be interpreted. Place-based approaches by nature require adaptation to local needs and findings from one context may not be generalizable to others.

## Conclusion

This study provides an example of how a place-based rural admission and training strategy will be coupled with an evaluation of workforce outcomes for the same region. Providing a baseline for the future evaluation of Deakin’s place-based RTS, the results demonstrate the factors that are associated with positive rural workforce outcomes for the footprint and support the place-based design of the RTS. The critical need for a broader range of rural specialty training pathways to be established to enable local rural retention in later post-graduate years is evident, and future research will evaluate the impact of these being established in the footprint.

## Data Availability

The data analyzed in this study is subject to the following licenses/restrictions: Ethics approval for access and use of this data is restricted to the purposes of this project. Requests to access these datasets should be directed to Jessica Beattie, j.beattie@deakin.edu.au.
